# A Luminosity

**Published:** 1862-07

**Authors:** 


**A LUMINOSITY.**—John Young Myrtle, M.D., F.R.C.P.E., and the rest of the A B C's, took the pains a "spell" ago to send a paper to the Medico-Chirurgical Society of Edinburgh, containing this very clear, concise, and precise prescription, in part: "As much hog's lard as a slice of fresh butter from the table." The *Boston Medical Journal* quotes the same without note, comment, exclamation, or explanation. The same indefiniteness, want of precision, is a very common defect in published recipes and medical prescriptions. It is better to throw every thing of the sort into the fire. Meanwhile, will the *Boston Medical Journal* tell its multitudinous readers what is the size of a "slice of fresh butter from the table"? Is it as large as a slice of salted butter from the same place? Is it as large or larger, or is it smaller, than a slice of butter from a tub or grocer's shelf? Is it as large as a piece of chalk? Is it as big as a turnip, or as the head of John Young Myrtle, with all the letters of the alphabet, and all the way from "Edinboro' town"? Within a few days, a life was nearly lost by a person following a newspaper "receipt," which printed a "tablespoonful" instead of a teaspoonful. No person should swallow any medicine from a newspaper direction, unless it has been submitted to an authorized druggist or an intelligent physician. And every editor owes it to his own defense, and to the welfare of his patrons, to publish no suggestion as to human health, without giving the authority; and not even then, unless the name of the person or publication has some claim on public credence.

